# Associations of Weight and Glucose Levels With Myasthenia Gravis: A Two‐Step, Two‐Sample Mendelian Randomization and Retrospective Case‐Control Study

**DOI:** 10.1002/brb3.71726

**Published:** 2026-08-31

**Authors:** Hai Lin, Jinrong Zhu, Xi Qu, Shenyi Lin, Shengqi Li, Yukai Wang, Jiaxuan Chen, Ruting Wei, Yanyi Pan, Xinran Li, Ruizi Xu, Suwen Huang, Yiyun Weng

**Affiliations:** ^1^ Department of Endocrinology The Third Affiliated Hospital of Wenzhou Medical University Wenzhou Zhejiang China; ^2^ Department of Neurology The First Affiliated Hospital of Wenzhou Medical University Wenzhou Zhejiang China; ^3^ The Second School of Medicine Wenzhou Medical University Wenzhou Zhejiang China; ^4^ The First School of Medicine, School of Information and Engineering Wenzhou Medical University Wenzhou Zhejiang China; ^5^ Renji College, Wenzhou Medical University Wenzhou Zhejiang China

**Keywords:** Mendelian randomization, myasthenia gravis, weight, glucose

## Abstract

**Background:**

Previous studies suggested links between obesity, glucose regulation, and autoimmune diseases, but the associations of weight and glucose levels with myasthenia gravis (MG) remain uncertain. We examined these associations and a potential indirect pathway through glucose levels.

**Objective:**

We investigated the associations of weight and glucose levels with MG risk using Mendelian randomization (MR) and a retrospective case‐control study.

**Methods:**

We conducted two‐sample MR analyses using genome‐wide association study summary statistics from European populations. The clinical component retrospectively included patients with MG and healthy controls and used propensity‐score matching and logistic regression.

**Results:**

The MR analyses suggested associations among genetically predicted weight, glucose levels, and MG risk. They also yielded a small indirect estimate through glucose levels that was opposite in direction to the direct and total effects. This opposing‐direction indirect estimate was sensitive to the inferential method and was not observed in the retrospective case‐control analysis. In the clinical analysis, MG cases had higher weight and lower glucose levels than controls, and both variables were associated with MG status.

**Conclusion:**

Two‐sample MR suggested that genetically predicted higher weight was associated with higher odds of MG, whereas genetically predicted higher glucose levels were associated with lower odds of MG. The retrospective case‐control analysis showed directionally similar associations for measured weight and fasting glucose, but the indirect pathway suggested by MR was not observed in the clinical analysis.

## Graphical abstract

In a two‐step, two‐sample Mendelian randomization framework, genetically predicted higher weight was associated with higher odds of myasthenia gravis (MG; OR = 1.67), whereas higher glucose levels showed an inverse association with MG (OR = 0.50). Genetically predicted higher weight was also associated with higher glucose levels (β = 0.06), and the MR analysis yielded a small opposing‐direction indirect estimate through glucose levels (indirect‐to‐total effect ratio = −8.68%). Consistent with these findings, in the propensity score‐matched case‐control cohort, MG cases exhibited higher weight but lower glucose levels than controls. The indirect pathway was not observed in the clinical analysis.

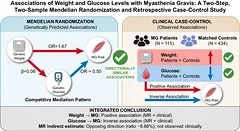

## 1 Introduction

Myasthenia gravis (MG) is an autoimmune disease that affects the postsynaptic membrane at the neuromuscular junction, typically characterized by fatigue‐induced muscle weakness with milder symptoms in the morning and exacerbation towards the end of the day (Gilhus et al. 2019). Among MG patients, approximately 80% test positive for antibodies to the acetylcholine receptor, while a minority exhibit antibodies to muscle‐specific kinase and low‐density lipoprotein receptor‐related protein 4. Additionally, around 5% of MG patients do not test positive for any known autoantibodies (Gilhus and Verschuuren 2015). In China, the incidence rate of MG is approximately 0.68 per 100,000 person‐years, with an in‐hospital mortality rate of 14.69 per 1000 admissions. There exists a certain sex disparity, with an incidence rate of 0.76 per 100,000 in females and 0.60 per 100,000 in males (Chen et al. 2020).

Numerous studies have highlighted a consistent link between high weight and increased risk of autoimmune diseases across different age groups. Obesity is associated with systemic inflammation and immune dysregulation, contributing to the development of autoimmune conditions such as multiple sclerosis, rheumatoid arthritis, and Type 1 diabetes (Marcus et al. 2022; Li et al. 2023; Gremese et al. 2014). These findings suggest that excess weight may play a broader role in autoimmune pathogenesis. In MG patients, dual‐energy X‐ray absorptiometry (DXA) studies have reported a higher prevalence of obesity and increased android and total fat mass, often accompanied by reduced lean mass (Chang et al. 2021). These MG‐specific body composition shifts suggest that higher weight may reflect a clinically meaningful metabolic load rather than body size alone. Supporting the relevance of metabolic burden in MG, an elevated triglyceride‐glucose index has been associated with increased risk of thymoma‐associated MG (Zhu et al. 2025), and higher weight has been linked to comorbidities such as obstructive sleep apnea in MG populations (Heo et al. 2019). Therefore, weight may serve as a simple and clinically accessible indicator of metabolic and immunologic burden in MG.

Research has revealed that glucose intolerance and impaired glucose regulation may occur in certain autoimmune conditions, such as multiple sclerosis (Wens et al. 2014). Both observational and epidemiological studies have suggested links between glucose dysregulation and MG. Clinical case‐control data showed that Type 2 diabetes was associated with MG (Liu et al. 2023), while a recent bidirectional MR analysis suggested an association between genetic liability to Type 1 diabetes and MG (Li et al. 2024). Furthermore, in experimental models, glucose dysregulation could worsen the severity of MG, and interventions targeting glucose dysregulation may mitigate its onset (Zhang et al. 2021; Cui et al. 2019). However, studies linking glucose levels to the severity of MG are mostly based on animal research, with limited clinical evidence. Numerous studies have also established a tight connection between overweight and glucose dysregulation.

Taken together, previous evidence suggests that weight and glucose regulation may be associated with MG, but their relationships and a potential indirect pathway through glucose levels remain insufficiently characterized. We therefore examined these associations using MR and a retrospective case‐control study. We hypothesized that higher weight would be associated with higher MG risk and that glucose levels might account for part of this association.

## 2 Materials and Methods

### 2.1 Study Design

**FIGURE 1:**
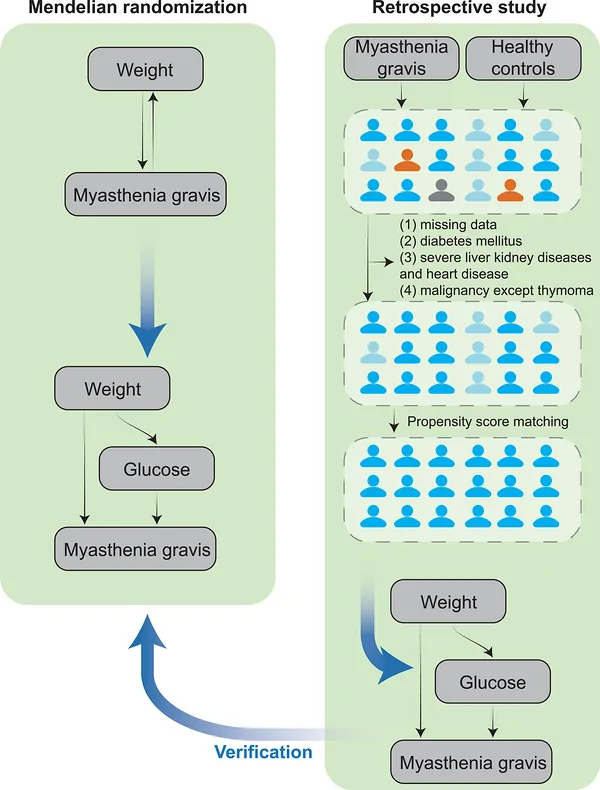
Flowchart for this study.

This study comprised two components: (1) two‐sample MR analyses using publicly available GWAS summary statistics and (2) a hospital‐based retrospective case‐control study of patients with MG and healthy controls. We first performed bidirectional MR analyses of weight and MG. We then used two‐step MR to estimate the association of genetically predicted weight with glucose levels and the association of genetically predicted glucose levels with MG risk, thereby evaluating a potential indirect pathway through glucose levels (Carter et al. 2021). The clinical component examined corresponding associations using measured weight and fasting glucose. Additional MR analyses evaluated the associations of genetically predicted weight with Type 1 and Type 2 diabetes and the associations of genetic liability to each diabetes‐related trait with MG risk (Figure 1).

### 2.2 Data Source

Because MG patients may exhibit altered body composition, including increased adiposity and reduced lean mass, BMI may not fully capture adiposity‐related metabolic burden in this population (Chang et al. 2021). Weight was therefore used as a pragmatic measure of overall body mass in the clinical analysis and as the primary MR exposure because a large GWAS with well‐powered instruments was available. However, weight cannot distinguish fat mass from lean mass, does not fully account for height‐ or sex‐related differences in body composition, and may be affected by fluid status. It should therefore not be interpreted as a diagnostic measure of obesity. Including height in the propensity‐score model reduced but did not eliminate this limitation. Fasting glucose was examined as a potential metabolic intermediate. In the clinical cohort, fasting glucose was measured in morning blood samples collected after an overnight fast.

This MR study relied on publicly available summary‐level GWAS datasets. As a primary analysis, the MG data were obtained from the largest available meta‐GWAS conducted in the US and Italy (1873 patients versus 36,370 age/sex‐matched controls, https://www.ebi.ac.uk/gwas/studies/GCST90093061) (Chia et al. 2022). Additionally, summary statistics for weight were derived from the MRC‐IEU database (461,632 participants, https://gwas.mrcieu.ac.uk/datasets/ukb‐b‐11842), while GWAS summary data for glucose were obtained from the GWAS Catalog platform (114,870 participants, https://www.ebi.ac.uk/gwas/studies/GCST90092819) (Richardson et al. 2022). The other GWAS data sources were detailed in Supplementary Table 1. The weight GWAS reported genetic associations in standardized units (SD), and the glucose GWAS provided effect estimates on an SD scale. Therefore, for continuous outcomes (weight and glucose levels), effect estimates are reported as β coefficients with 95% confidence intervals, reflecting SD change in the outcome per 1‐SD increase in genetically predicted exposure. Binary outcomes (MG, Type 1 diabetes, Type 2 diabetes) are interpreted on the log‐odds scale and are reported as odds ratios (ORs).

In the case‐control segment, we incorporated data from hospitalized patients diagnosed with MG at the First Affiliated Hospital of Wenzhou Medical University between June 2016 and June 2022, along with data from healthy individuals from the same hospital in 2013. We also excluded cases with missing data, implausible data values, diabetes mellitus, severe liver and kidney diseases, severe heart conditions, and malignant tumors other than thymoma. Ultimately, this study included 134 MG patients and 2008 healthy controls. At the same time, in order to ensure the comparability of data between MG patients and the healthy controls, we utilized propensity score matching to reduce potential confounding effects. To minimize potential confounding by body size, height was included in the propensity score matching model. Some cases could not be matched to four controls within the caliper and were retained with fewer matched controls. After matching, 115 patients with MG and 434 healthy controls were included. The clinical component was a single‐center retrospective case‐control study, and weight and fasting glucose were measured at a single time point during hospitalization. These measurements may have been influenced by disease status, nutritional intake, acute stress, or previous treatment; therefore, their temporal relationship with MG could not be established.

### 2.3 Statistical Analysis

We conducted two‐sample MR analyses to estimate the associations among genetically predicted weight, glucose levels, and MG (Davey Smith and Hemani 2014). Eligible instrumental single‐nucleotide polymorphisms (SNPs) were selected using prespecified quality‐control criteria. Firstly, significant SNPs associated with exposure were chosen based on genome‐wide significance (*p‐*value < 5 × 10^−8^) and minor allele frequency (MAF) > 0.01. Subsequently, we performed linkage disequilibrium pruning using European reference samples from the 1000 Genomes Project (*R* ^2^< 0.001, window size = 10,000 kb). Finally, the exposure data were harmonized with the outcome data using the harmonization function. Instrument strength was evaluated using the approximate per‐SNP F statistic, *F* = (β_exposure/SE_exposure)^2^, calculated from the exposure summary statistics for each SNP retained after harmonization (Burgess et al. 2011). An *F* statistic < 10 was prespecified as indicating weak instrument strength (Staiger and Stock 1997). Across the eight MR directions, 7–485 SNPs were retained. None had *F* < 10, and the overall minimum F statistic was 29.61, providing no evidence of weak instrument strength according to the prespecified threshold (Supplementary Table 2). To evaluate the robustness of the MR estimates, we performed MR‐PRESSO (Mendelian randomization pleiotropy residual sum and outlier) analyses with 10,000 simulated distributions per direction and a fixed random seed, including the global test, the outlier test, and the distortion test after outlier removal. Where outliers were identified, outlier‐corrected estimates were reported alongside the uncorrected estimates. We also performed leave‐one‐out analyses, in which the IVW estimate was recalculated after excluding each SNP in turn. These sensitivity analyses are reported with their raw *p‐values* and were not included in the primary BH‐FDR correction family. We used inverse‐variance weighted (IVW), MR‐Egger, and weighted‐median methods (Bowden et al. 2016). IVW was the primary estimator, while MR‐Egger and weighted‐median estimates were used as complementary sensitivity analyses. All causal estimates for continuous traits were scaled to a 1‐SD increase in genetically predicted exposure, consistent with the original GWAS definitions. To assess heterogeneity, we calculated Cochran's Q value and *p*‐value. In addition, we performed an MR‐Egger intercept to detect directional horizontal pleiotropy (Bowden et al. 2015). A Cochran's Q test *p‐*value < 0.05 was considered evidence of heterogeneity across instrumental SNPs. For pleiotropy assessment, a significant MR‐Egger intercept (*p* < 0.05) was taken to indicate the presence of directional horizontal pleiotropy. MR analyses were based on the R package “TwoSampleMR” (Hemani et al. 2018). To control for multiple testing, the Benjamini–Hochberg false discovery rate (FDR) correction was applied to the eight prespecified primary IVW analyses. Both raw and FDR‐adjusted *p‐values* for all eight primary IVW analyses are reported in Supplementary Table 2; relevant adjusted values are also reported in the results. This approach was implemented using the base *R* function p.adjust (method = “fdr”). Associations with *p*_FDR < 0.05 were considered statistically significant. The application of FDR correction ensures that the false positive rate was adequately controlled across multiple MR comparisons. We performed two‐step MR to evaluate whether the effect of genetically predicted weight on MG risk was mediated through glucose levels. First, we estimated the causal effect of weight (X) on glucose levels (M) using IVW MR, yielding β_XM. Second, we estimated the causal effect of glucose levels (M) on MG (Y) using IVW MR, yielding β_MY. The indirect (mediated) effect was calculated as β_indirect = β_XM×β_MY. The total effect (β_XY) of weight on MG was obtained from univariable MR. The direct effect was calculated as β_direct = β_XY‐β_indirect. The indirect‐to‐total effect ratio was calculated as β_indirect/β_XY. Because the indirect and total effects had opposite signs, the resulting ratio was negative. The negative sign was interpreted as indicating an opposing‐direction indirect effect, and the ratio was not interpreted as a conventional mediation proportion. Two approaches were used to quantify statistical uncertainty. First, the standard error was approximated by the delta method: SE (β_indirect) = √((β_MY^2·SE_XM^2) + (β_XM^2·SE_MY^2)), and a two‐tailed Wald‐type *p*‐value was derived under asymptotic normality. Second, 95% confidence intervals were additionally estimated by non‐parametric bootstrap with 5,000 resamples. A two‐sided empirical bootstrap *p*‐value was calculated as twice the smaller of the proportions of bootstrap estimates on either side of zero. Inference from the bootstrap and delta methods was reported in parallel because the product term may have an asymmetric sampling distribution and the two methods yielded slightly different conclusions in this study. Binary outcomes were interpreted on the log‐odds scale and reported as odds ratios (ORs), while continuous traits were interpreted per 1‐SD increase in the genetically predicted exposure, consistent with the original GWAS definitions. All procedures were implemented in R version 4.0.3 (R Foundation for Statistical Computing, Vienna, Austria).

In the clinical component, variables showing between‐group differences at *P* <0.10, specifically age and height, were included in a 1:4 propensity‐score matching procedure with a caliper of 0.2. Shapiro–Wilk tests indicated non‐normal distributions for the continuous variables; these variables were therefore compared using nonparametric tests, while categorical variables were compared using chi‐square tests. Logistic regression was used to examine the associations of weight and fasting glucose with MG status, and restricted cubic splines were used to explore potential nonlinear associations. Clinical mediation analysis was performed using nonparametric bootstrapping with 5000 resamples in the R package mediation (version 4.5.0) and SPSS 26.0 (IBM, Armonk, NY, USA). No additional covariates were included in the mediation model to limit overfitting given the sample size. The clinical mediation analysis was considered exploratory because the measurements were cross‐sectional and their temporal ordering could not be established.

## 3 Results

### 3.1 Bidirectional Association Between Weight and Myasthenia Gravis

**FIGURE 2:**
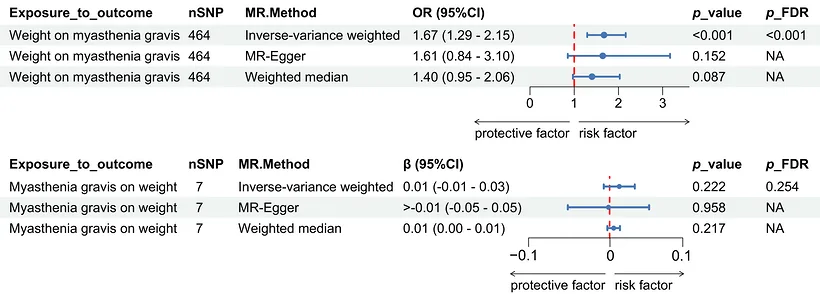
Bidirectional mendelian randomization of weight and MG.

In bidirectional MR analyses, genetically predicted higher weight was associated with higher odds of MG (464 SNPs, OR = 1.67; 95% CI, 1.29‐2.15; *p* < 0.001, *p*_FDR < 0.001), whereas in the reverse direction, genetic liability to MG was not associated with weight (Figure 2, Supplementary Figure 1).

### 3.2 Weight, Glucose Levels, and Myasthenia Gravis: Two‐Step MR Mediation Analysis

**FIGURE 3:**
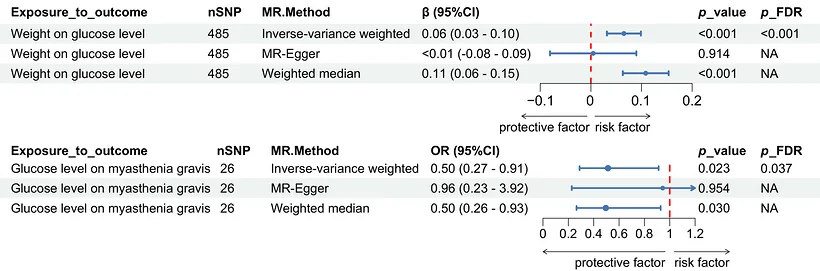
Mendelian randomization of glucose levels in the association between weight and MG.

To explore the association between glucose levels and MG and the possible mediating role of glucose in the effect of weight on MG risk, we performed two‐step, two‐sample MR analyses. Genetically predicted higher weight was associated with higher glucose levels (485 SNPs, β = 0.06; 95% CI, 0.03‐0.10; *p* < 0.001, *p*_FDR < 0.001), whereas genetically predicted higher glucose levels were associated with lower odds of MG (26 SNPs, OR = 0.50; 95% CI, 0.27‐0.91; *p* = 0.023, *p*_FDR = 0.037) (Figure 3). The indirect effect of weight on MG risk through glucose levels acted in the opposite direction to the total effect. In the two‐step MR mediation analysis, genetically predicted weight showed a positive total effect on the risk of MG. Genetically predicted higher weight was also associated with higher glucose levels. In contrast, genetically predicted higher glucose levels were associated with a lower risk of MG. The estimated indirect effect of weight on MG through glucose levels was negative (β_indirect = −0.044; nonparametric bootstrap 95% CI, −0.093 to −0.007, *p* = 0.018; delta‐method 95% CI, −0.089 to 0.001, *p* = 0.053). The indirect estimate was opposite in direction to the direct and total effects, but its statistical interpretation was sensitive to the inferential method. After accounting for this indirect pathway, the remaining direct effect of weight on MG remained positive (β_direct = 0.555). The indirect‐to‐total effect ratio was approximately −8.68%. The negative sign indicates that the indirect effect was opposite in direction to the total effect; therefore, this signed ratio was not interpreted as a conventional mediation proportion (Supplementary Figure 2).

### 3.3 Associations Among Diabetes‐Related Traits, Weight, and Myasthenia Gravis

**FIGURE 4:**
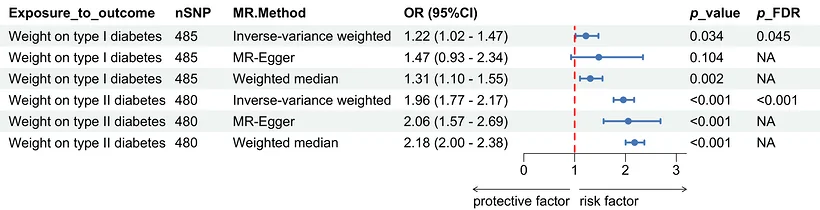
Mendelian randomization of weight and diabetes‐related traits.

**FIGURE 5:**
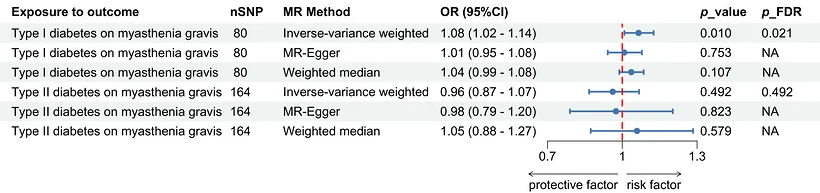
Mendelian randomization of diabetes‐related traits and MG.

After observing an association between weight and glucose levels, we further explored the association between weight, diabetes‐related traits, and MG. Through MR analysis, we explored that genetically predicted higher weight was associated with Type 1 diabetes (485 SNPs, OR = 1.22; 95% CI, 1.02‐1.47; *p* = 0.034, *p*_FDR = 0.045) and Type 2 diabetes (480 SNPs, OR = 1.96; 95% CI, 1.77–2.17; *p* < 0.001, *p*_FDR < 0.001) (Figure 4). Meanwhile, genetically predicted Type 1 diabetes was associated with higher odds of MG (80 SNPs, OR = 1.08; 95% CI, 1.02–1.14; *p* = 0.010, *p*_FDR = 0.021), whereas no significant association was observed between Type 2 diabetes and MG risk (Figure 5).

To assess the robustness of the primary IVW findings, we performed MR‐PRESSO and leave‐one‐out sensitivity analyses for all eight MR directions (Supplementary Table 2; Supplementary Figure 5). MR‐PRESSO global tests indicated some evidence of heterogeneity or horizontal pleiotropy in seven of the eight directions; outliers were detected in six directions, and after outlier removal, the corrected estimates remained in the same direction and retained the same significance classification as the primary estimates in all directions in which outliers were identified. Leave‐one‐out analyses showed no reversal of the effect direction in any direction, and the *p* < 0.05 classification was stable in six of eight directions. In the glucose→MG analysis, rs6972516 produced the largest absolute change in the point estimate; however, exclusion of rs560887 was the only one of 26 iterations that yielded a non‐significant IVW estimate (*p* = 0.068). In the weight→T1DM analysis, exclusion of rs28366156 yielded a non‐significant IVW estimate in one of 485 iterations (*p* = 0.051). These two associations were therefore interpreted with caution.

Cochran's Q tests suggested heterogeneity across instrumental SNPs in most analyses. Therefore, we used a random‐effects IVW model as the main estimator and performed weighted median and MR‐Egger as sensitivity analyses. MR‐Egger intercept tests did not provide evidence of directional horizontal pleiotropy in most associations, except for the T1DM→MG analysis; residual pleiotropy therefore cannot be excluded (Supplementary Figure 4).

### 3.4 Characteristics of Enrolled Patients

We used the data of MG patients from the First Affiliated Hospital of Wenzhou Medical University and the data of healthy individuals for a case‐control study. Before matching by propensity score, there were significant differences in age, weight, and glucose levels between MG patients and healthy controls (*p* < 0.001, *p* < 0.001, *p* < 0.001). The median age of MG patients was 48.00 (35.00‐61.00) and that of healthy controls was 42.00 (34.00–52.00); the median weight of MG patients was 59.50 (53.00–66.00), and that of healthy controls was 56.50 (52.00–62.50); the median glucose levels of MG patients were 4.70 (4.30–5.30), and that of healthy controls was 5.40 (5.20–5.70) (Table 1).

**TABLE 1:** Characteristics of MG patients and healthy controls.

Variables	Before propensity score matching			After propensity score matching		
	MG (*n* = 134)	HCs (*n* = 2,008)	*p* value	MG (*n* = 115)	HCs (*n* = 434)	*p* value
Age (years)	48.00 (35.00–61.00)	42.00 (34.00–52.00)	< 0.001	48.00 (37.00–61.00)	48.00 (36.00–57.00)	0.361
Sex (male, n (%))	57 (42.54)	825 (41.09)	0.741	49 (42.61)	216 (49.77)	0.172
Height (cm)	163.00 (158.00–169.00)	161.50 (156.00–168.50)	0.064	163.00 (159.00–169.00)	162.50 (158.00–169.13)	0.779
Weight (kg)	59.50 (53.00–66.00)	56.50 (52.00–62.50)	< 0.001	59.00 (54.00–66.00)	57.50 (52.88–63.00)	0.035
Glucose (mmol/L)	4.70 (4.30–5.30)	5.40 (5.20–5.70)	< 0.001	4.60 (4.30–5.20)	5.40 (5.20–5.70)	< 0.001

Abbreviation: HCs, healthy controls.

Variables with *p‐values* < 0.1 for the comparison of differences between groups were regarded as possible confounding variables. After the propensity score matching with a 1:4 caliper of 0.2 for height and age, there were significant differences in weight and glucose levels between MG patients and healthy controls (*p* = 0.035; *p* < 0.001). After matching, the median weight of MG patients was 59.00 (54.00–66.00), and that of healthy controls was 57.50 (52.88–63.00); the median glucose levels of MG patients were 4.60 (4.30–5.20), and that of healthy controls was 5.40 (5.20–5.70) (Table 1). There were significant differences in weight and glucose levels between MG patients and healthy controls. Compared with the healthy controls, the weight level of the MG group was higher, but the glucose levels were lower than those of the healthy controls. At the same time, in the violin plot, we compared their distribution. Compared with the healthy controls, the weight level of the MG group was higher, and the glucose level was lower, and the difference was statistically significant (*p* = 0.035 and *p* < 0.001, respectively) (Supplementary Figure 3).

### 3.5 Association Between Weight, Glucose Levels and Myasthenia Gravis

To explore the association of weight and glucose levels with MG, we used logistic regression. In the univariate logistic regression results, the association of weight and glucose levels with the risk of MG could be observed. Weight was associated with higher odds of MG (OR = 1.04; 95% CI, 1.02–1.07; *p* = 0.002), while glucose levels were associated with lower odds of MG (OR = 0.47; 95% CI, 0.34–0.65; *p* < 0.001); After adjustment for possible confounding effects of sex, the association still existed between weight (OR = 1.08; 95% CI, 1.05–1.12; *p* < 0.001), glucose levels (OR = 0.47; 95% CI, 0.34–0.66; *p* < 0.001) and MG (Table 2).

**TABLE 2:** Association between weight, glucose levels and MG.

Variables	Univariate analysis		Multivariate analysis	
	OR (95% CI)	*p* value	OR (95% CI)	*p* value
Weight (kg)	1.04 (1.02–1.07)	0.002	1.08 (1.05–1.12)	< 0.001
Glucose (mmol/L)	0.47 (0.34–0.65)	< 0.001	0.47 (0.34–0.66)	< 0.001

Abbreviations: CI, confidence interval; MG, myasthenia gravis; OR, odds ratio.

Multivariable model adjusted for sex.

**FIGURE 6:**
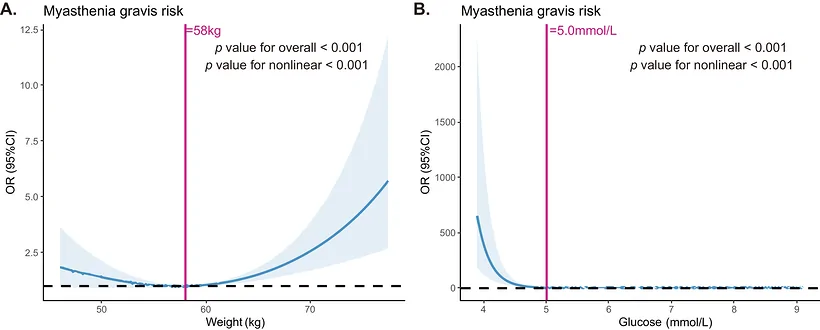
Nonlinear association between glucose levels, weight, and myasthenia gravis. (A) The association between weight and MG risk showed a U‐shaped pattern; (B) MG risk was significantly increased when the blood glucose level was lower than 5.0 mmol/L.

To explore the association between glucose levels and weight levels and the risk of MG under the assumption of a nonlinear relationship, we used restricted cubic splines. The non‐linear association assumption of both was significant (*p* < 0.001, *p* < 0.001), and the overall association was also significant (*p* < 0.001, *p* < 0.001). MG risk followed a U‐shaped association with weight, with the lowest risk observed around 58 kg. The risk of MG was significantly increased when the glucose levels were below 5.0 mmol/L; the association appeared to plateau above 5.0 mmol/L, with the estimated OR approaching unity (Figure 6).

We then examined the corresponding indirect pathway in the clinical data. However, the indirect effect of weight on MG through glucose was not statistically significant (β_indirect = −0.002; 95% CI, −0.015–0.007; *p* = 0.588) (Table 3).

**TABLE 3:** Mediation analysis of glucose levels in the association between weight and MG.

Variables	Mediation analysis	
	Effect (95% CI)	*p* value
Weight to glucose	0.003 (−0.008–0.014)	0.585
Weight and glucose to MG		
Weight	0.043 (0.016–0.069)	0.002
Glucose	−0.771 (−1.101–−0.441)	< 0.001
Direct effect	0.043 (0.016–0.069)	0.002
Indirect effect*	−0.002 (−0.015–0.007)	0.588

Abbreviation: CI, confidence interval.

Effects are reported as regression coefficients on their original scales. The weight to glucose path is on the fasting glucose scale (mmol/L), and the MG outcome model coefficients (weight to MG, glucose to MG, direct effect) are on the log‐odds scale. The indirect effect is expressed as the product of these scales.

^*^95% CIs and p value for indirect effect estimated using percentile bootstrap with 5000 resamples.

The MR and clinical analyses showed directionally similar associations: higher weight was associated with higher odds of MG, whereas higher glucose levels were associated with lower odds of MG. The MR analysis further suggested a small opposing‐direction indirect effect of weight on MG through glucose levels. However, this indirect pathway was not statistically significant in the clinical analysis (*p* = 0.588). Because the two components differed in ancestry, data source, measurement, and study design, their directional agreement should not be interpreted as cross‐population replication.

## 4 Discussion

In this study, MR and case‐control methods were used to explore the association between weight, glucose, and the risk of MG. The MR analyses suggested that genetically predicted weight and glucose levels were associated with MG risk. Among them, glucose was associated with lower odds of MG, while weight was associated with higher odds of MG. We also found that the indirect effect of weight on MG risk through glucose levels was opposite in direction to the direct and total effects. Notably, the indirect effect through glucose was small in magnitude compared with the total effect, indicating that most of the weight‐MG association was driven by the direct pathway. In mediation analysis, a competitive or suppressive mediation pattern occurs when the indirect effect through a mediator operates in the opposite direction to the direct effect, resulting in a partial attenuation of the total effect (Zhao et al. 2010). In this study, the positive direct and total estimates and the negative indirect estimate were compatible with a suppressive or competitive mediation pattern. As a hypothesis, this finding may be interpreted as a potential “glucose paradox” whereby higher genetically predicted glucose levels were associated with a lower MG risk despite the overall detrimental effect of excess weight. One hypothesis is that weight and glucose reflect different metabolic or immunologic processes; however, these mechanistic interpretations remain speculative. Within the MR framework, mediation effects are decomposed into total, direct, and indirect components under instrumental variable assumptions (Carter et al. 2021; Burgess et al. 2015). The presence of opposing direct and indirect effects does not violate MR assumptions but instead reflects directionally heterogeneous causal pathways, as described in methodological studies of MR‐based mediation analysis. Directionally similar associations were observed in the clinical data. The association between higher weight and increased MG risk, as well as between higher glucose levels and reduced MG risk were observed. However, the opposing‐direction indirect effect was not observed in the clinical data. In addition, genetically predicted weight was associated with diabetes‐related traits, and genetic liability to Type 1 diabetes was associated with MG risk.

In the sensitivity analyses, Cochran's Q tests indicated significant heterogeneity across instrumental SNPs. This finding suggests that the SNP‐specific causal estimates may vary, which could arise from biological diversity in variant mechanisms, weak instrument variability, or the presence of some invalid instruments. Importantly, in large‐scale GWAS with a substantial number of genetic instruments, Cochran's Q statistics may be highly sensitive and frequently detect heterogeneity even when the overall causal direction remains stable. To account for this, we relied primarily on the IVW random‐effects model, which provides more conservative inference under heterogeneity, and performed complementary analyses using weighted median and MR‐Egger regression. Consistent with this concern, MR‐PRESSO global tests also indicated evidence of heterogeneity or horizontal pleiotropy in seven of the eight directions; in the six directions with detected outliers, the corrected estimates retained the same direction and *p* <0.05 classification as the primary IVW estimates (Supplementary Table 2). The distortion test was significant for MG→weight and weight→T2DM, but outlier correction did not change the direction or the significance classification of these associations. This indicates that outlier removal changed the magnitude of these estimates even though their direction and significance classification were unchanged. Leave‐one‐out analyses showed no reversal of the effect direction in any direction; however, the *p* < 0.05 classification changed for glucose→MG and weight→T1DM after removal of a single SNP (rs560887 and rs28366156, respectively) (Supplementary Figure 5), and these associations were therefore interpreted with caution. MR‐Egger intercept tests showed no evidence of directional horizontal pleiotropy for most associations, except for the T1DM→MG analysis, which was treated as exploratory (Supplementary Figure 4). Nevertheless, given the evidence of heterogeneity, our MR results, particularly those with large Q statistics, should be interpreted with caution.

The results of this study are consistent with the results of existing studies on the situation of diabetes‐related traits (Liu et al. 2023; Li et al. 2024), weight level in the population of MG patients, and the association with the risk of MG (Chang et al. 2021; Chen et al. 2025). Through MR and case‐control analyses, this study examined the associations of these factors with MG. Moreover, the directions of the associations observed in the European MR analyses were consistent with the clinical observations in Chinese patients; however, differences in ancestry, data source, measurement, and study design limit direct extrapolation between the two populations. The directional similarity should not be interpreted as direct replication or cross‐population confirmation.

The MR analysis yielded an opposing‐direction indirect estimate through glucose levels, whereas the corresponding indirect estimate was not statistically significant in the retrospective clinical analysis. Importantly, in the clinical dataset, weight and glucose were collected during hospitalization, and thus may be influenced by disease status, nutritional intake, acute stress, or previous treatment, making the temporal ordering of exposure–mediator–outcome unclear. Because the prediagnostic and pretreatment measurements were unavailable, reverse causation cannot be excluded. Therefore, the clinical mediation results should be considered exploratory and hypothesis‐generating rather than causal. Prospective studies with prediagnostic or pretreatment measurements are needed to evaluate the pathway suggested by the MR analysis.

A key strength of this work is the evidence from a hospital‐based case‐control analysis and two‐sample MR. While the clinical analysis addresses real‐world phenotypes, the MR component evaluates genetically predicted effects that are less prone to confounding and reverse causation. There are also many limitations and shortcomings in this study. For example, the MR analysis was conducted using European GWAS data, whereas the clinical analysis involved a Chinese population. Such ancestry differences may introduce potential heterogeneity that could influence the results. However, directional similarity across these two components does not establish cross‐ancestry replication, and further studies in ancestrally diverse populations are needed. Secondly, the clinical component was a small, single‐center retrospective case‐control study based on cross‐sectional measurements, which limits precision and representativeness. Specifically, after propensity score matching, only 115 MG cases and 434 controls were included in the analysis. This limited sample size may reduce the statistical power to detect modest associations or mediation effects and may affect the representativeness of the clinical findings. Further evaluation in larger, multicenter prospective cohorts is warranted. At the same time, in the MR study, we could not guarantee that GWAS participants are completely homogeneous, because there is a lack of demographic data for GWAS participants, such as age and sex. This may affect the analysis results. Although the sensitivity analyses did not suggest that horizontal pleiotropy fully explained the main findings, residual pleiotropy cannot be excluded. This study mainly discussed the association of weight, glucose regulation imbalance and the risk of MG, but did not investigate the underlying mechanism, which needs further research. Future studies should aim to validate these findings in larger, multi‐ethnic cohorts and through prospective longitudinal designs to better establish temporal causality. In addition, integrative omics approaches, including metabolomics and immunogenomics, could help elucidate the biological mechanisms linking weight, glucose regulation, and autoimmune activity in MG. The present findings do not establish that modifying glucose levels would prevent MG or improve clinical outcomes. Another limitation of the retrospective case‐control analysis is that MG cases were recruited between 2016 and 2022, whereas the healthy control group was derived from a health examination database in 2013. This mismatch in calendar time may introduce systematic bias, because laboratory platforms, assay calibration, reference ranges, clinical workflows, and electronic record systems may change across years, particularly for fasting glucose measurements, which constitute a primary variable in this study. Although both groups originated from the same hospital and we applied propensity score matching to improve comparability, residual bias due to temporal differences cannot be excluded. Therefore, the observational findings should be interpreted as supportive rather than causal, and future validation using controls recruited within the same time window or prospective cohorts is warranted.

A large number of studies have shown that high weight is an important risk factor for the imbalance of glucose regulation (Narayan et al. 2007; di Bonito et al. 2017). DXA‐based studies have reported that MG patients exhibit notable body composition changes, including a higher prevalence of obesity, increased android fat deposition, and higher total body fat percentage, along with reductions in regional skeletal muscle mass compared with controls (Chang et al. 2021). However, the mechanism of the effect of glucose regulation imbalance on MG patients is still inconclusive. The existing researches mainly believed that it might be achieved by affecting the glucose metabolism mode of peripheral blood immune cells and regulating adaptive and innate immune responses. Studies have observed that in MG patients, different immune cell subtypes show different patterns of glucose metabolism. The expression levels of key enzymes involved in glycolysis and HIF‐1α were significantly higher in B cells, dendritic cells, Tregs, CD 4^+^CD25^−^T cells, Th1, and Th17 cells of MG patients. Meanwhile, in MG patients, the glycolytic level and glycolytic capacity of B cells and dendritic cells were significantly higher than those of CD 8^+^ T cells, CD 4^+^ T cells, and their subsets (Li et al. 2020). Further, diabetes‐related traits could regulate adaptive and innate immune responses. In animal experiments, diabetes promoted adaptive and innate immunity and exacerbated the clinical symptoms of experimental autoimmune MG rats (Zhang et al. 2021). Additionally, in animal experiments, oral metformin could reduce the onset of experimental autoimmune MG, and this effect was accompanied by a significant reduction in circulating autoantibody levels (Cui et al. 2019). These immune‐cell and animal findings provide possible mechanistic hypotheses but do not establish the mechanism underlying the human MR or clinical associations observed here.

Therefore, weight and glucose levels were associated with MG in the MR and clinical analyses, and the MR analyses suggested a small opposing‐direction indirect effect through glucose levels. These findings support the hypothesis that weight and glucose regulation are associated with MG risk. Moreover, this pattern may help explain the higher weight and lower glucose levels observed in MG patients compared with healthy controls. The biological and clinical relevance of this indirect estimate remains uncertain.

## 5 Conclusion

In both the genetic and clinical analyses, weight and glucose levels were associated with the risk of MG. Higher weight was associated with higher odds of MG, whereas higher glucose levels were associated with lower odds of MG. A small opposing‐direction indirect effect through glucose levels was suggested by the MR analysis, although this pattern was not observed in the clinical analysis. These findings warrant further evaluation in prospective studies and ancestrally diverse populations.

## Author Contributions

**Hai Lin**: data curation, formal analysis, writing – original draft, writing – review and editing, investigation, validation, visualization, software, methodology. **Jinrong Zhu**: data curation, formal analysis, writing – original draft, writing – review and editing, investigation, validation, visualization, software, methodology. **Xi Qu**: data curation, formal analysis, writing – original draft, writing – review and editing, investigation, validation, visualization, software, methodology. **Shenyi Lin**: data curation, formal analysis, investigation, validation, writing – review and editing. **Shengqi Li**: data curation, investigation, writing – review and editing. **Yukai Wang**: data curation, investigation, writing – review and editing. **Jiaxuan Chen**: data curation, writing – review and editing, investigation. **Ruting Wei**: data curation, writing – review and editing, investigation. **Yanyi Pan**: data curation, writing – review and editing, investigation. **Xinran Li**: data curation, investigation, writing – review and editing. **Ruizi Xu**: data curation, writing – review and editing, investigation. **Suwen Huang**: conceptualization, methodology, writing – review and editing, project administration, resources, supervision. **Yiyun Weng**: conceptualization, methodology, writing – review and editing, project administration, resources, supervision.

## Funding

The authors have nothing to report.

## Ethics Statement

The study was approved by the Institutional Ethics Committee Review Committee of the First Affiliated Hospital of Wenzhou Medical University (No. KY2023‐R124) and was conducted in accordance with the Declaration of Helsinki.

## Conflicts of Interest

The authors declare no conflict of interest.

## Supporting information




**Supplementary Information**: brb371726‐sup‐0001‐SuppMat.docx

## Data Availability

The GWAS sources presented in the study are included in the article/Supplementary Material. Further inquiries can be directed to the corresponding authors.
