# Supplementary material for: Associations of Weight and Glucose Levels With Myasthenia Gravis: A Two‐Step, Two‐Sample Mendelian Randomization and Retrospective Case‐Control Study
Source: Brain Behav. 2026 Aug 31;16(9):e71726. doi: 10.1002/brb3.71726 (PMC13527523; doi:10.1002/brb3.71726)
Supplement: Supplementary file 1 [file BRB3-16-e71726-s001.docx]

ORIGINAL RESEARCH

**Associations of Weight and Glucose Levels with Myasthenia Gravis: A Two-Step, Two-Sample Mendelian Randomization and Retrospective Case-Control Study**

**Catalogs**

**Supplementary Tables**	3

Supplementary Table 1. GWAS data source of this study	3

**Supplementary Table 2.** Instrument strength and quality metrics for the eight Mendelian randomization analyses. 4

**Supplementary Figure**	7

Supplementary Figure 1. Linear association between body weight and myasthenia gravis	7

Supplementary Figure 2. Two-step MR estimates for the indirect pathway from weight to myasthenia gravis through glucose levels	8

Supplementary Figure 3. Differences in weight and glucose levels between MG patients and healthy controls. 9

Supplementary Figure 4. Heterogeneity and pleiotropy assessment for MR analysis	10

Supplementary Figure 5. Leave-one-out analyses for the eight Mendelian randomization directions. 11

| Factors | Dataset | Population | Sample size | Sex | Year | Data source  (PMID) | Unit/Scale |
| --- | --- | --- | --- | --- | --- | --- | --- |
| Myasthenia gravis | ebi-a-GCST90093061 | US and Italian | 1,873/36,370 | NA | 2022 | 35074870 | Binary (OR) |
| Weight | ukb-b-11842 | European | 461,632 | Males and Females | 2018 | MRC-IEU | SD |
| Glucose levels | ebi-a-GCST90092819 | European | 114,870 | NA | 2022 | 35213538 | SD |
| Type 1 diabetes | ebi-a-GCST90014023 | European | 18,942/501,638 | NA | 2021 | 34012112 | Binary (OR) |
| Type 2 diabetes | ebi-a-GCST90018926 | European | 38,841/451,248 | NA | 2021 | 34594039 | Binary (OR) |

**Supplementary Table 1.** GWAS data source of this study.

**Supplementary Table 2.** Instrument strength and quality metrics for the eight Mendelian randomization analyses.

**Panel A. Instrument strength and IVW estimates.**

| **MR direction (exposure→outcome)** | **nSNP** | **F, min** | **F, median** | **F, max** | **F < 10, n** | **IVW β (95% CI)** | **IVW *P*** | **FDR *P*** |
| --- | --- | --- | --- | --- | --- | --- | --- | --- |
| Weight→MG | 464 | 29.82 | 47.85 | 1173.50 | 0 | 0.51 (0.25 to 0.77) | 9.60×10^-5 | 3.84×10^-4 |
| Weight→Glucose | 485 | 29.82 | 47.90 | 1173.50 | 0 | 0.06 (0.03 to 0.10) | 2.23×10^-4 | 5.95×10^-4 |
| Glucose→MG | 26 | 30.71 | 38.20 | 537.15 | 0 | -0.70 (-1.31 to -0.09) | 0.0234 | 0.0374 |
| MG→Weight | 7 | 30.64 | 41.25 | 60.66 | 0 | 0.01 (-0.01 to 0.03) | 0.2219 | 0.2536 |
| T1DM→MG | 80 | 29.87 | 49.32 | 8073.95 | 0 | 0.07 (0.02 to 0.13) | 0.0103 | 0.0205 |
| T2DM→MG | 164 | 29.61 | 47.75 | 1066.63 | 0 | -0.04 (-0.14 to 0.07) | 0.4920 | 0.4920 |
| Weight→T1DM | 485 | 29.82 | 47.90 | 1173.50 | 0 | 0.20 (0.02 to 0.38) | 0.0339 | 0.0452 |
| Weight→T2DM | 480 | 29.82 | 47.88 | 1173.50 | 0 | 0.67 (0.57 to 0.78) | 1.06×10^-37 | 8.44×10^-37 |

**Panel B. Heterogeneity, pleiotropy and MR-PRESSO.**

| **MR direction (exposure→outcome)** | **Cochran's Q (df)** | **Q *P*** | **MR-Egger intercept (SE)** | **Egger *P*** | **MR-PRESSO global *P*** | **Outliers, n** | **Outlier-corrected β (95% CI)** | **Corrected *P*** | **Distortion *P*** |
| --- | --- | --- | --- | --- | --- | --- | --- | --- | --- |
| Weight→MG | 581.33 (463) | 1.47×10^-4 | 0.0006 (0.0056) | 0.9177 | 2.00×10^-4 | 0 | NA | NA | NA |
| Weight→Glucose | 873.55 (484) | 9.29×10^-25 | 0.0011 (0.0007) | 0.1436 | <0.0001 | 3 | 0.07 (0.04 to 0.10) | 3.52×10^-6 | 0.6033 |
| Glucose→MG | 56.24 (25) | 3.38×10^-4 | -0.0322 (0.0317) | 0.3197 | 6.00×10^-4 | 1 | -0.61 (-1.18 to -0.04) | 0.0480 | 0.6460 |
| MG→Weight | 87.79 (6) | 8.72×10^-17 | 0.0051 (0.0089) | 0.5933 | <0.0001 | 2 | 0.01 (-0.00 to 0.01) | 0.1318 | <0.0001 |
| T1DM→MG | 256.40 (79) | 1.31×10^-20 | 0.0317 (0.0107) | 0.0042 | <0.0001 | 5 | 0.08 (0.03 to 0.13) | 0.0016 | 0.6400 |
| T2DM→MG | 178.70 (163) | 0.1895 | -0.0010 (0.0078) | 0.8962 | 0.1893 | 0 | NA | NA | NA |
| Weight→T1DM | 1974.36 (484) | 1.13×10^-178 | -0.0034 (0.0040) | 0.3901 | <0.0001 | 9 | 0.22 (0.10 to 0.33) | 2.06×10^-4 | 0.7762 |
| Weight→T2DM | 2930.21 (479) | <0.001 | -0.0008 (0.0023) | 0.7121 | <0.0001 | 41 | 0.77 (0.70 to 0.85) | 1.46×10^-64 | 0.0191 |

**Caption:** The approximate per-SNP F statistic was calculated as F = (β_exposure / SE_exposure)^2 from the exposure summary statistics for each SNP retained after harmonization. Instruments with F < 10 were considered weak; none of the retained instruments had F < 10. IVW β estimates are on the log-odds scale for binary outcomes and on the SD scale for continuous outcomes, per 1-SD increase in the genetically predicted exposure; odds ratios are reported in the main text and Figures 2-5. Cochran's Q (with degrees of freedom), MR-Egger intercept and MR-PRESSO global tests are reported as sensitivity analyses without inclusion in the FDR family; FDR P values were computed only for the eight pre-specified IVW analyses. MR-PRESSO was performed with 10,000 random distributions and a fixed random seed for each direction. NA in Panel B indicates that no outlier SNP was detected for that direction; outlier-corrected estimates and distortion tests were therefore not applicable.

**Abbreviations:** CI, confidence interval; FDR, false discovery rate (Benjamini-Hochberg correction across the eight pre-specified IVW analyses); IVW, inverse-variance weighted; MR, Mendelian randomization; PRESSO, Pleiotropy REsidual Sum and Outlier; SE, standard error; SNP, single-nucleotide polymorphism; T1DM, type 1 diabetes; T2DM, type 2 diabetes.

**Supplementary Figure 1.** Linear association between body weight and myasthenia gravis.

**Supplementary Figure 2.** Two-step MR estimates for the indirect pathway from weight to myasthenia gravis through glucose levels

**Caption:** Two-step MR analysis of the indirect pathway from genetically predicted weight to MG risk through glucose levels. Total effect was estimated by IVW result of weight on MG. Indirect effect was calculated as the product of the IVW estimates from weight to glucose (path a) and glucose to MG (path b). Direct effect was derived as total effect minus indirect effect. Uncertainty in the indirect estimate was evaluated using the delta method and 5,000 nonparametric bootstrap resamples. The bootstrap interval excluded zero, whereas the delta-method interval included zero. The indirect-to-total effect ratio was −8.68%. The negative sign indicates that the indirect estimate was opposite in direction to the positive total effect; therefore, this signed ratio should not be interpreted as a conventional proportion mediated. This opposing-direction pattern is compatible with competitive mediation.

**Abbreviations:** CI, confidence interval; IVW, inverse-variance weighted; MR, Mendelian randomization; MG, myasthenia gravis.

**Supplementary Figure 3.** Differences in weight and glucose levels between MG patients and healthy controls.

**Caption:** Violin plots show the distributions of (A) weight (kg) and (B) fasting glucose levels (mmol/L) in patients with MG (n = 115) and healthy controls (n = 434). The central dashed line and flanking dotted lines indicate the median and quartiles, respectively. *p* values were obtained from nonparametric between-group comparisons.

**Abbreviations:** HCs, healthy controls; MG, myasthenia gravis.

**Supplementary Figure 4.** Heterogeneity and pleiotropy assessment for MR analysis.

**Caption:** Cochran’s Q statistic and its corresponding *p* value (Q_*p*) were used to assess heterogeneity among SNP-specific estimates. The MR-Egger intercept, standard error, and corresponding *p* value (*p*) were used to assess directional horizontal pleiotropy. Cochran’s Q tests indicated heterogeneity in seven of the eight directions, whereas the MR-Egger intercept test indicated evidence of directional horizontal pleiotropy only for T1DM→MG. The displayed *p* values are unadjusted sensitivity-analysis *p* values.

**Abbreviations:** MR, Mendelian randomization; SE, standard error.

**Supplementary Figure 5.** Leave-one-out analyses for the eight Mendelian randomization directions.

**Caption:** Each panel shows the leave-one-out random-effects inverse-variance weighted (IVW) estimates for one of the eight MR directions: Weight→MG, MG→Weight, Weight→Glucose, Glucose→MG, Weight→T1DM, Weight→T2DM, T1DM→MG, and T2DM→MG. Each point represents the IVW estimate with its 95% confidence interval obtained after excluding the indicated SNP (x-axis, index of the omitted SNP); the red horizontal line indicates the IVW estimate based on all retained SNPs; the dashed grey line indicates the null (zero effect); red points denote the SNP whose exclusion produced the largest absolute change in the estimate. Estimates are expressed per 1-SD increase in the genetically predicted exposure, on the log-odds scale for binary outcomes.

**Abbreviations:** CI, confidence interval; IVW, inverse-variance weighted; MG, myasthenia gravis; MR, Mendelian randomization; SNP, single-nucleotide polymorphism; T1DM, type 1 diabetes; T2DM, type 2 diabetes.
